# Psychiatric trainees’ experiences of workplace violence: qualitative analysis

**DOI:** 10.1192/bjb.2024.6

**Published:** 2025-04

**Authors:** Lauren Fowler, Alisha Vara, Lillian Ng

**Affiliations:** University of Auckland, Auckland, New Zealand

**Keywords:** Education and training, mental health services, qualitative research, risk assessment, violence

## Abstract

**Aims and method:**

We aimed to explore experiences of workplace violence in a New Zealand sample of psychiatric trainees and to identify barriers to achieving safe practice and ways of enhancing workplace safety. In a qualitative study, we used interpretive description to inform and design in-depth exploration of participants’ experiences. We interviewed 12 psychiatric trainees. Data were analysed using reflexive thematic analysis.

**Results:**

There were three main themes: (a) violence as ‘part of the job’, leading to a culture of silence; (b) empowering trainees to address a sense of learned helplessness; and (c) conflict embedded within the unique nature of psychiatry.

**Clinical implications:**

Organisation-led systems-based procedures are instrumental in promoting workplace safety. Specific measures include peer-based support and implementing clear, tailored safety protocols, particularly for situations of crisis assessment. Training should include culturally focused education with specific guidance to mitigate violence.

All health professionals should feel safe in their workplace, to enable them to care optimally for patients and protect their own well-being.^1,2^ Psychiatric trainees may be at higher risk of threats from patients, compared with doctors training in other specialties.^1,3,4^ Rates of physical assault on psychiatric trainees range between 36 and 56%, with verbal threats ranging from 48 to 96%.^2,5–8^ These figures may not surprise, given the high-acuity environments, which are often ill-equipped to manage patients’ needs, as well as co-existing substance use and socioeconomic disadvantage that are frequently seen in patients in the mental health setting.^1,6,9^ This is not to say that people with mental illness are inherently violent.^9^ The risk of violent assaults is higher for trainees, compared with consultants.^1,10^ Therefore it is important to understand the experiences of psychiatric trainees in relation to workplace violence, uncover barriers to achieving safe practice and explore ways to improve safety. Violence in the workplace includes verbal, psychological, physical and sexual abuse. Psychiatric registrars perceive violence of any type as stressful.^11,12^ Many are unaware of formal avenues to report incidents.^12^ One study found that just 9% of trainees who suffered verbal or physical abuse received post-incident support.^2^ Trainees have reported inadequate safety training, with some perceiving they were to blame for events.^2,3,6,11–13^ Violence may be a precursor to leaving the profession, compounding staff shortages in psychiatry. Protecting staff, particularly trainees, should be a top priority.^14^

In a literature review we did not find any studies providing an in-depth understanding of experiential workplace violence from the sole perspective of psychiatric trainees. Contextual factors are important, including cultural elements. For example, trainees’ experiences in New Zealand may be more similar to those in Australia and the UK compared with countries such as the USA, where gun violence is prominent.^15^ Literature exploring workplace violence among psychiatric trainees is predominantly limited to retrospective surveys which fail to comprehensively explore the context of violence.^1,2,5–8,12,13,16,17^ Most research is more than 20 years old and practice has changed considerably over this time.^1,5,6,8,10,18^ In this study, we aimed to explore psychiatric trainees’ experience of workplace violence and guide future recommendations for safe practice.

## Method

This study has been conducted according to standards for reporting qualitative research.^19^ We utilised an inductive qualitative framework to capture an in-depth conceptualisation of trainees’ experiences of workplace violence.^20^

### Study design

The research team comprised a psychiatric trainee and two psychiatrists who work in academia and the clinical setting. We used an interpretive description approach to the research question,^21^ acknowledging our clinical lens and declaring our investment in promoting workplace safety for psychiatric doctors. To provide a more objective perspective, we employed an independent researcher to co-code and facilitate the development of themes. We conducted a literature search in consultation with a specialist librarian on two occasions (1 October 2021 and 17 April 2023) in MEDLINE (Ovid), Embase (Ovid), PsycInfo (Ovid) and Scopus databases, within a 20-year period. The search terms were: ‘psychiatr*’ used in conjunction with ‘training’, ‘internship*’, ‘interns’, ‘intern’, ‘registrar*’, ‘clerkship*’, ‘trainee*’or ‘residen*’ and combined with ‘violen*’, ‘workplace violence’, ‘assault*’, ‘abuse*’, ‘abusive’, ‘abusing’, ‘threat*’, ‘physical’ ‘violence’, ‘safe*’ or ‘hazard*’. We devised an interview schedule, which was discussed with a psychiatrist and a Māori cultural advisor working in an acute clinical setting. The questions were predominantly open ended, allowing for thorough exploration of trainees’ experiences with regard to violent encounters. Demographic data, including clinical experience and workplace safety training, were acquired at the beginning of the interview. The interview was piloted on a senior psychiatric trainee and modified to optimise interview flow. The interview questions are listed in Supplementary Table 1, available at https://doi.org/10.1192/bjb.2024.6.

### Study participants and data collection

Qualitative interviews were conducted in a purposive sample of psychiatric trainees from the northern region of New Zealand. All trainees that were associate members of the Royal Australian and New Zealand College of Psychiatrists with a minimum of 6 months of training were invited via email to participate by a training administrator. Between September and December 2022, the first author (L.F.) conducted semi-structured individual interviews in person or by video-link with trainees that expressed an interest in participating. Face-to-face interviews were used where possible, to facilitate rapport. Where trainees expressed a preference for audio-visual link, Zoom or Microsoft Teams were utilised. The interviews were audiotaped using a digital recorder (interview duration 29–58 min). All participants gave written consent to take part in the research. Prior to the interview, participants were provided with an abbreviated topic guide to encourage thoughtful reflection on the day.

### Ethical considerations

The authors assert that all procedures contributing to this work comply with the ethical standards of the relevant national and institutional committees on human experimentation and with the Helsinki Declaration of 1975, as revised in 2008. All procedures involving human subjects/patients were approved by the University of Auckland Human Participants Ethics Committee (UAHPEC24035).

### Data analysis

Interviews were transcribed verbatim by the first author, which allowed for familiarisation with the data, consistent with reflective thematic analysis.^20,22^ Member checking was offered to ensure transcript accuracy and allow for additional reflections: two participants provided further information. Data were stored in NVivo version 14. There were three successive rounds of independent coding: (a) codes and initial themes were discussed (by L.F. and A.V.); (b) themes were further identified (L.F. and L.N., alongside the independent researcher); (c) themes were refined in consultation with all authors, and consensus drawn with the independent researcher. The online tool ‘Miro’ was used to aid the process of analysis and document reflections and discussions sequentially, in keeping with an audit trail.

## Results

In total, 12 psychiatric registrars (4 male and 8 female) who had worked across a variety of clinical settings participated. Of these, most (75%) had completed mandatory workplace safety training. Three central themes were identified: theme 1: violence as ‘part of the job’, leading to a culture of silence; theme 2: empowering trainees to address a sense of learned helplessness; and theme 3: conflict embedded within the unique nature of psychiatry. Further quotations relating to the themes are shown in the Supplementary material.

### Theme 1: violence as ‘part of the job’, leading to a culture of silence

#### Acceptance and normalisation of workplace violence by trainees and the organisation

All participants viewed violence as an inherent occupational hazard, ‘just part of the job’. Violence was normalised and accepted. The topic evoked emotive responses and a sense of frustration:

‘I didn't feel it was worth escalating it because I didn't think anyone would actually do anything about it.’

Participants felt guilty when violent incidents occurred. There was a high emotional toll as trainees stated they became desensitised to violence over time, with an incremental impact on their clinical practice and their personal lives:

‘[ … ] the job wears you down generally. Patients say some hurtful and outrageous things [ … ] the cumulative effect is a little bit soul destroying and I've noticed that my capacity outside of work to deal with other things [ … ] has probably suffered.’

Participants recognised detrimental effects on well-being, including feelings of resentment, anger and anxiety:

‘That was the final straw. I was quite bitter and jaded toward psychiatry. I felt a bit disappointed, why do I [ … ] put this effort in if this is what I have to deal with?’

#### Feeling exposed by limitations on resources and supports

Accepting violence as an occupational hazard led to participants being resigned to restricted resources. These were listed as paucity of staff and limited access to security and personal safety devices such as alarms. They reported being vulnerable to violence because they felt unsupported. This set up a vicious cyclical relationship: by accepting violence as the norm, trainees were underprepared for dangerous encounters. Ultimately, the problem was exacerbated by difficulties with staff retention and burnout:

‘We end up with more staffing issues, more overworked, which means we have less time for self-care and to rectify it. It's a vicious cycle.’

Variation in staffing experience and difficulties establishing professional relationships were cited as limitations in fostering a safe working environment, particularly in the on-call setting. Participants identified interpersonal discord in working with less experienced clinicians and those with more complacent attitudes towards violence. Trainees advocated for education about workplace violence for all clinicians:

‘Some clinicians can be quite challenging. They have this sometimes judgmental, critical stance on the patient. This can aggravate the problem or provoke them.’

Working in acute mental health services was described as ‘running at full stretch’. All emphasised practical measures such as being prepared to assess patients, securing police attendance at assessments and good-quality triage:

‘I was anxious, there wasn't the backup so I got left quite vulnerable.’

#### The on-call environment is inherently fraught with danger

Tolerance towards violence was pronounced in the on-call setting. The unfamiliar environment evoked anxiety in trainees, particularly when visiting patients at home:

‘Reviewed him in his house, way out with no phone signal in the middle of nowhere. Diffusely unwell. Had a shotgun on the premises. At one point was pretty agitated and went and showed us his shotgun.’

The often skeletal staff presence in the crisis setting was perceived as unsafe and illogical, and after-hours work was viewed as volatile. Trainees frequently conducted solo assessments in the emergency department during nightshifts, owing to being ‘understaffed’ or ‘not having support staff overnight or on site’. Most expressed concern about assessing patients on their own and one reported that a staff member had refused to accompany them:

‘[ … ] this often happens in crisis context where I might be asked to go see someone on my own [ … ].’

Participants identified difficulties establishing rapport with patients with fluctuating mental state, substance misuse and emotional dysregulation in the acute setting as contributory to danger:

‘[ … ] we're dealing with patients who can be more hypervigilant, feel more threatened, fearful. All those factors might make someone more at risk of retaliatory violence, trying to protect themselves.’

### Theme 2: empowering trainees to address a sense of learned helplessness

This theme comprised learned helplessness, self-awareness of cultural factors and safety as a core component of training. Trainees often felt invalidated and poorly equipped to advocate for their personal safety.

#### Learned helplessness and blame within a medico-legal context

Participants described exposure to unsafe situations and powerlessness to prioritise their own safety, despite holding significant clinical responsibility. As trainees, they attempted to articulate concerns yet felt unheard by senior staff and managers, becoming reluctant to communicate their apprehension. Two refrained from seeking support, fearing their concerns would be unacknowledged. The majority of trainees perceived having little control over their tasks, time or support given and unable to set their own parameters. Trainees sought to be active in the decision-making process but perceived they lacked authority to be so. On occasions, six participants had felt under duress to work. As their experience grew, so too did their confidence to speak out:

‘[ … ] our nursing colleagues usually have a lot more experience, that does sometimes mean that they're a little bit more gung ho [ … ].’

‘I've had several home visits where I was a much less experienced registrar and I questioned how necessary they were at the time. We kind of got browbeaten into doing them anyway [ … ].’

#### Self-awareness in culturally safe practice

Participants highlighted challenges skilfully navigating interactions where cultural elements are at play. Trainees acknowledged historical injustices in the background of a doctor–patient power dynamic, particularly for indigenous Māori. Trainees emphasised avoiding assumptions and approaching cultural interactions with inquisitive naivety. This included recognising their own limitations and biases, acknowledging patients as experts and respecting their *mana* (Māori term for agency). A collaborative approach was seen as imperative and achievable by utilising cultural supports:

‘[ … ] even thinking about the historical traumas that have happened to Māori and when they're in the throes of a psychosis and put in the establishment that may evoke a lot of that intergenerational trauma or distrust [ … ].’

Cultural supports were a scarce resource. Trainees recognised the need to develop skills in cultural safety:

‘[ … ] knowing what the culture sensitivities and being culturally appropriate around how to approach certain topics, how to approach the interview, involvement of the cultural team.’

#### Forewarned is forearmed: managing workplace violence as a core component of training

Participants expressed feeling overworked and underprepared to deal with violence. Education on workplace violence needed to be in the early stages of training, as junior doctors had little exposure prior to embarking on that vocational pathway. Trainees spoke of gaining knowledge by ‘a baptism of fire’:

‘I think just how new and naive I was, that I could handle that on my own. I hadn't learned the lesson yet, but I shouldn't have learnt it this way [ … ].’

Trainees sought changes in working conditions, linking burnout to poor performance. They recognised that being alert to danger was compromised by long hours, high workload and being unable to process incidents:

‘[ … ] the workload is so immense, that something shocking will happen and you are expected to just brush it off and move on to the next patient.’

Trainees wanted access to clear safety protocols and more education to promote agency in asserting these protocols:

‘There's no consensus on it [ … ] the safety precautions vary wildly.’

### Theme 3: conflict embedded within the unique nature of psychiatry

Trainees described many attributes inherent to psychiatric practice that increase the risk of violence.

#### Resistance to psychiatry shaped by stigma and historical abuse, evoking violence

Four trainees identified negative societal perceptions of psychiatry, shaped by historical wrong-doings. They were mindful of stigma attached to psychiatry as evoking intense emotions. Psychiatric diagnoses were often met with resistance:

‘[ … ] psychiatry itself has this enormous stigma [ … ] you are essentially telling somebody they are mentally unwell, there is a lot of natural resistance and it stirs up really intense feelings, not just in patients, also in the family members of patients and a wider circle.’

#### In the line of fire: restrictive contexts invoking combative response

The legal framework of psychiatry was perceived as restrictive and at times coercive. Trainees observed patients adopting a combative stance in response to the constraints placed on them, which damaged rapport and was a precursor to violence. Four commented that other specialties do not routinely treat patients against their will:

‘[ … ] the Mental Health Act is coercive in nature. That goes against the usual approach you take with patient centred care.’

Interactions with police were also found by several to be challenging, with discord between actual practice and protocols, contributing to interprofessional tension:

‘The police had refused to bring him in because they didn't think they had grounds. On site the guy was brandishing a knife surrounded by four police officers in stab vests and the police tried to convince me that I needed to go into the middle and talk to him.’

#### The emergency department is not fit for purpose

Trainees highlighted the lack of purpose-built clinical spaces to assess patients. The needs of psychiatric patients were seen to be neglected as they languished in the emergency department owing to lack of more appropriate environments, designed with physical health as a priority. The high-stimulus environment of the emergency department lacked privacy for discussing sensitive topics and security was often suboptimal. Trainees recognised that environments not fit for purpose exacerbated agitation and increased the risk of violent encounters:

‘[ … ] it's very high stimulus. It's a very difficult place to be and so this is often where I find people are more agitated.’

## Discussion

In this study, we explored experiential workplace violence in a sample of trainee psychiatrists in the northern region of New Zealand. They depicted a pervasive acceptance of workplace violence, highlighting a culture of silence in our specialty. Our results indicate a need to empower trainees to advocate for their safety in a healthcare system that, like many others internationally, is under immense strain. The unique nature of psychiatry, against a backdrop of stigma and abuse, sets a scene for difficult interactions between staff and patients. We do not believe these challenges to be insurmountable, with trainees emphasising an array of practical measures to promote workplace safety. The first, albeit most challenging, step is acknowledging this as a serious issue that needs urgent attention.

Our study confirms that workplace violence is indeed ‘part of the job’, a finding that is prevalent throughout the literature.^2,3,6,12,16,17^ Similar to other studies, we found that normalisation perpetuates a sense of self-blame.^6,11,16^ Beyond just recognition of this phenomenon, interventions to foster safety should be prioritised. Comprehensive, systematic and focused education imparting specific skills and strategies to mitigate violence can provide a solid foundation, which can be reinforced and built on as training progresses. The reported deficit in current understanding of workplace violence, with trainees calling for more tailored education, is consistent with other studies.^1,3,5–8,12^ Despite literature supporting the need to implement early training in workplace safety, our study demonstrates that trainees believe this does not occur locally, in part because they are not aware of training opportunities.^3^ Trainees highlighted emphasis on safety in every situation and adequate preparation for assessments as areas for improvement.^5^

We have identified the importance of trainees utilising a collaborative approach in cultural interactions to help mitigate violence. The New Zealand Medical Council has highlighted the importance of examining power relationships that impede best outcomes for Māori *whai ora* (‘a person who is the subject of care, assessment and treatment processes in mental health’).^23,24^ This is true of other indigenous and minority ethnic groups internationally where mental health legislation is used significantly more, exacerbating social exclusion.^25^ Mindful of achieving equitable health outcomes, we suggest a sharp focus on supporting trainees to upskill in ways that are culturally safe, which includes reflecting on personal biases and checking cultural assumptions. Adopting culturally safe practice will engender a non-threatening environment for vulnerable groups, including Māori. Practically, this can be achieved by improving availability of cultural supports and including mandatory culturally focused training in the psychiatry curriculum.

A sense of solidarity was highlighted, underlining the importance of peer support to improve safety. Trainees felt that by turning to their fellow registrars, they had more confidence to assert their safety needs and to retrospectively support decision-making for future practice. Similarly to other studies, there was clear vulnerability in being ‘a newbie’, a phase in which trainees felt at greater risk owing to inexperience.^10^ They suggested ways to utilise the peer dynamic, especially between junior and advanced trainees.

Psychiatry is fraught with stigma and historical institutional abuse internationally. Fear of maltreatment is an unsavoury yet expected consequence. New Zealand is no different. For example, the infamous Lake Alice Hospital casts a shadow on psychiatry, as electroconvulsive therapy and painful paraldehyde injections were used on children under the guise of psychiatric treatment.^26^ The present study underscores the importance of recognising and sensitively working with emotional trauma associated with psychiatric stigma. It highlights difficulties in current local legal frameworks that can feel coercive to both staff and patients and eerily reminiscent of historical abuse.

In keeping with international literature, crisis assessments, particularly in the emergency department, were cited as violence-prone.^5,13,27^ This study adds to knowledge on trainees’ perceptions of home visits in the on-call setting. It highlights trainees’ beliefs that home visits in the on-call setting in the northern region of New Zealand are potentially unsafe and overused, particularly when police are absent. This is concerning, given our findings of learned helplessness and difficulties garnering support when concerns are raised. We believe the utility of home visits in the crisis setting requires further examination, given their unpredictable nature. Balancing this with difficulties faced by patients is an important consideration in not perpetuating societal inequity for those who cannot easily access care.

Although local protocols exist to promote staff safety, these need to be more accessible, enforced and discussed with trainees at specific stages of training to support them in articulating their needs. Safety is rooted in a shared understanding of risk. Therefore, procedures to enhance safety must involve multiple stakeholders, such as clinicians, patients, families and cultural advisors. Workplace violence is one of the most stressful events for psychiatric trainees and can lead to distressing psychological consequences.^11^ Trainees are part of the multidisciplinary team and adapt to increased levels of clinical responsibility and leadership during the course of their training. Protecting trainees may avoid burnout and facilitates sustainable practice and staff retention.^28^ We emphasise the need to support trainees to work in safer environments and to process emotional distress following sentinel events.

### Strengths and limitations

By utilising reflexive thematic analysis, we interpreted detailed accounts from participants specifically to explore context as it relates to violence. This qualitative approach using interpretive description is in keeping with psychiatry's focus on complex experiential encounters.^29^ The dual clinician–researcher role permits a lens to examine the contextual elements of violence in this setting.^30^ The first author's ‘insider’ status as a psychiatric trainee enabled establishment of rapport to facilitate thick description.^22,31^ The absence of power imbalance may have enhanced the quality of responses, by allowing trainees to speak freely about their encounters to someone with shared experiences. Other study strengths included member checking and steps to enhance the robustness of the analysis by employing an independent researcher for coding and objective adjudication of themes. Unfortunately, no Māori or Pacific trainees participated in the study, and their participation might have provided a more diverse perspective on workplace violence, particularly cultural barriers. We do not presume our sample to be representative of all psychiatric trainees. We encapsulated a range of views across different clinical settings, although opinions are limited to the local setting. Trainees who experienced adverse encounters may have been more motivated to take part.

### Recommendations and future directions

Our results demonstrate the need to balance service provision with staff safety. Measures including peer-based supports and implementing clear safety procedures within the workplace are practical starting points. Training should include cultural education focused on mitigating violence. Given the specific challenges faced in psychiatry, strategies to minimise risk, including the availability of more resources such as cultural support, should be prioritised.^24^ We endorse the current revision of New Zealand mental health legislation, which considers patients’ perspectives and incorporates a rights-based approach to assessment and treatment.

Future research on perspectives of allied health professionals and managers would be valuable, to explore whether trainees’ experiences are viewed in a similar light. There may also be benefit in researching trainees in other contexts, internationally, to compare experiences and identify helpful measures to collectively address workplace violence from an organisational perspective.

## Supporting information

Fowler et al. supplementary material 1 Fowler et al. supplementary material

Fowler et al. supplementary material 2 Fowler et al. supplementary material

## Data Availability

The data and analytic codes that support the findings of this study, together with the topic guide, are available from the corresponding author, L.N., on request. The data are not publicly available as they contain information that could compromise the privacy of research participants.

## About the authors

**Lauren Fowler** is an honorary lecturer in the Department of Psychological Medicine, University of Auckland, Auckland, New Zealand. **Alisha Vara** is an honorary senior lecturer in the Department of Psychological Medicine, University of Auckland, Auckland, New Zealand. **Lillian Ng** is a senior lecturer in the Department of Psychological Medicine, University of Auckland, Auckland, New Zealand.
