# Supplementary material for: Psychiatric trainees’ experiences of workplace violence: qualitative analysis
Source: BJPsych Bull. 2024 Mar 4;49(2):132–7. doi: 10.1192/bjb.2024.6 (PMC12014366; doi:10.1192/bjb.2024.6)
Supplement: Fowler et al. supplementary material 1 — Fowler et al. supplementary material [file S2056469424000068sup001.docx]

| Introduction | As you know, psychiatrists work with acutely unwell patients which can lead to challenging and sometimes dangerous clinical encounters. Did this factor into your decision when choosing psychiatry as a career? Why or why not? |
| --- | --- |
| One | Can you tell me about a time you’ve experienced workplace violence, whether this be physical, psychological, sexual or verbal?  Or can you tell me about a time you have felt you were at risk of being assaulted within the workplace? |
| Two | Can you tell me about any other times you have felt threatened or in danger at work? How did this affect you? |
| Three | Can you describe a time you felt you were psychologically unsafe at work? |
| Four | Psychiatric trainees are at higher risk of workplace violence compared to other specialties. Why do you think this is? |
| Five | How can safety be improved for psychiatric trainees with regard to workplace violence? |
| Six | If assaulted or threatened what support did you seek out following the incident? Explain. |
| Seven | What cultural factors do you consider when attempting to optimise workplace safety?  What measures would enhance safety when working with Māori Tangata whai ora and their whānau? |
| Eight | What do you think contributes to unsafe practice as a psychiatry trainee? What barriers do you see as achieving safe practice? |
| Nine | What resources or training have you come across that have been helpful in enhancing your safety in the workplace? |
| Ten | Do you think being a psychiatric registrar is a safe occupation? Why or why not. |
