# Supplementary material for: Psychiatric trainees’ experiences of workplace violence: qualitative analysis
Source: BJPsych Bull. 2024 Mar 4;49(2):132–7. doi: 10.1192/bjb.2024.6 (PMC12014366; doi:10.1192/bjb.2024.6)
Supplement: Fowler et al. supplementary material 2 — Fowler et al. supplementary material [file S2056469424000068sup002.docx]

**Supplementary Material: Quotations**

**Theme One: Violence as ‘part of the job’ leading to a culture of silence**

| **Subtheme** | **Quote** |
| --- | --- |
| **Acceptance and normalisation of workplace violence by trainees and the organisation** | *"It's just part of the job."*  *“And I think it just largely is just accepted as part of the job.”*  *“The nature of the job I guess.”*  *“And it's part of our jobs.”*  *"It’s a hard one because... and again I’m trying not to normalise it which I feel like everyone kind of does.”*  *“So I kind of… it's one of the weird things, if you've been dealing with stuff for long enough you kind of change the gauge of what's acceptable.”*  *“We need to stop normalising it.”*  *“I think there's always a level of inherent risk in this job.”*  *“But this really blasé attitude to a history of violence against staff is pervasive.”*  *“’I’m gonna rape you’ – you hear all of that stuff reasonably regularly. So I think the psychological impact is cumulative. And I think even if you're someone like me who doesn't fall apart when it happens, I think the heaviness of it weighs on you.”*  *“But other types of assault like verbal is very common because sometimes we are kind of used to it.”*  *“Yes I love the theory of psychiatry but I can’t do this job for the rest of my life and have to deal with that and have to be on edge."*  *“But I think we probably suppress a lot.”*  *“But it’s kind of hard not to just normalise this because you know these patients are unwell.”*  *“I guess sometimes I feel like if I bring up a safety concern, or if I say I don't really feel comfortable with this, it's sometimes brushed under the rug a little bit.”*  *“I think the barriers are basically our attitude toward violence. Sometimes we tolerate it. We shouldn't tolerate it.”*  *“I mean, I think in the moment, it certainly had a negative impact on my well-being. I don't think I ever felt unsafe in terms of my physical safety, but my mental well-being and emotional well-being were certainly impacted.”*  *“On my general well-being? Oh it wrecked my mental health previously.”*  *“And I think the CNM was like ‘oh its mental health, it’s just part of the job’. And I got quite cross with him because that attitude. I was really amazed that that was almost a callous disregard for well-being. And actually it isn’t part of the job. It's something that could potentially be a risk that we try and mitigate but if it happens, it should be considered serious.”*  *“So I guess that kind of leads to like system factors, which I think we strangely can tolerate things that no other service would tolerate. I think it is related to the population that we serve, but I do wonder if we allow too much to happen.”*  *“So I think that would help for things to be taken more seriously. And that yeah, even if people have had these experiences in the past, to not normalise them. Because even I think that even if you sort of feel alright at the time, especially with the peer dynamic or the pressure, you know, to just you know, be – I think it does, people go back in their minds to think about it and I think it can't not have some sort of negative effect.”*  *“I think the hate speech incident really jaded me.”*  *“And I also didn't feel it was worth escalating it because I didn't think anyone would actually do anything about it.”*  *“I guess perhaps there is an element of once you are engaged in that a lot and you do experience, particularly patient violence, I do wonder over time, whether that might wear me down and whether I’ll get a bit sick of it.”*  *“So I sort of felt like I didn’t do as good by the patient as I should have.”*  *“I feel like once we get involved, it's kind of expected that we'll just put up with a lot more unsafe behaviour, just to get the assessment type thing. I don't really know what the difference is to be honest.”*  *“We're kind of a bit more used to it. Desensitised to it I guess.”*  *“And then I guess, like the institutional stuff - do we maybe need to look at how we, what kind of stuff we tolerate within our services? And how maybe that culture can be addressed? Because yeah, that's going to be more system wide.”*  *“And I think you start to just normalise and your alertness for danger goes down because it's the kind of like just getting through the day getting the admin side of things, all the forms done, because I think I'm seeing the person and I'm thinking I've got three CAR forms to write because they've been booked back to back.”*  “*What I think we need to do more importantly, is have a culture where we say that staff safety is actually the priority. It's the priority over service delivery. It's the priority over timing. It's the priority over what we can and can't do yada yada yada. And that needs to be the overarching culture and under that culture would be a framework for dealing with situations, particularly situations that are difficult to control, like out in the community kind of assessments.”* |
| **Feeling exposed by limitations on resources and supports** | *"This is what creates burnout and creates poor retention of the staff and will exacerbate the problem."*  *"We end up with more staffing issues, more overworked, which means we have less time for self-care and to rectify it. So it's kind of a vicious cycle."*  *“I said previously, there's a couple of members of one of the acute teams locally that I won't do a home visit with.”*  *“It’s one of the reasons I’m leaving as well. I just don’t want to have to deal with that each day. It makes me kind of on edge.”*  *“I think for as long as I can remember and I assume it's getting worse is the poor funding. The lack of staff, the lack of resources, staff burnout, you know all those things?”*  *“Maybe I was anxious that there wasn't the backup so I got left quite sort of, I guess vulnerable.”*  *“So there needs to be a whole, quite robust system of triaging for these kinds of patients. And at the moment, as far as I can tell, there's not at all it's just like the Wild West.”*  *“Training of like, you know, more thorough training of nursing staff to recognise early warning signs to actually help direct the team.”*  *“I have no personal alarm. I know there is something on the door. But when you, I just feel that you know, naturally if there's something that's yeah, it'd be very difficult to quickly get to an alarm or open the door and things like that. It would be extremely tricky.”*  *“One time I was assessing a forensic client and the nurse who I was with, without any reason that I could determine, just sort of left the room halfway through the interview.”*  *“So now I'm having a stand up argument with the head of security at the hospital, because they're understaffed as well. And I started to think, psychiatry should actually have its own security, it should have its own personnel that we can call on for this sort of stuff.”*  *“Because I imagine it does contribute to the attrition rate for registrar's leaving the specialty on some level. Or a barrier for people going into the specialty and it’s a specialty that desperately needs more people. I know my friends who are doctors that don’t work in mental health couldn’t think of anything worse than working in mental health because of they perceive it as a dangerous occupation.”*  *“And I think the lack of consistent support from other staff members."*  *“I mean, there's just a general shortage of people working in mental health. So I appreciate that that's an issue. It's a much wider issue isn't it? Attracting people into mental health so that we can actually staff our rosters? For doctors and nurses and allied staff, we're barely able to do that at the moment.”*  *“There was no support. There was no security available.”*  *“And the colleague I was with, I no longer do home visits with because there's been a couple of similar incidents where he said something fucking stupid and things have escalated. And this guy dysregulated whilst manic. He's previously had a history of violence. He stood up he started becoming quite threatening and we left very quickly.”*  *“So I either need to trust the person I’m with or I need to trust that the environment can respond.”*  *“Yeah. I know the ED charge nurse has work to do, but I don’t think she should have walked away and left me to it.”*  *“I was there with new key workers like a social worker and a nurse, both of them really green and new and so the assurance I got prior to seeing this guy was from people with no experience whatsoever.”*  *“So no consensus of standards, poorly trained staff, new staff with no experience and not being counterbalanced with more experienced staff.”*  *" I was just kind of hauled out there without knowing much about the situation. I went immediately from one review to another, so home visit to home visit and I was handed his notes in the car by the nurse on call or whatever so I had very little preparation time for it."*  *“I think I've only had one occasion where a person was quite agitated and then the person stood up and took a step towards me. And the nurse sort of didn't do anything. And that was his decision of, you know, he wanted to try de-escalate, but it felt to me like oh, now this person is within arm's reach of me and could assault me and I wasn't protected. That was the only time that I didn't feel... Yeah, that was one of the times that I felt a bit unsafe.”*  *“And there's always obviously a nurse with you, but sometimes I find your threshold and the nurses threshold can be a bit different.”*  *"And I think we could be doing so much more to make it safer, but that would slow the service down. And the service is already running at full stretch. And so any slowing down of the service is unacceptable apparently."*  *"Mental Health Services kind of owning up to the big holes in the system. The fact that we do bend rules often and then providing training for the fact that we bend rules, would be much better than pretending we don't bend rules and giving us kind of...poor overall safety training."*  *“So I think that would help for things to be taken more seriously. And that yeah, even if people have had these experiences in the past, to not normalise them.”*  *“But it didn't get continued because they didn't want to fund security. And I think that that in itself is a testament to how little shit they give about risk. Because actually, if you're going to massively improve your service, based on the cost of security, on a weekend or an evening, it’s a minimal cost. You're going to drive up job satisfaction, you’re making people safer, so I think it was one of those things where it's just daft.”*  *“I think the barriers is just the difficulties we continue to have with staff shortages and funding issues and things that limit us from a system point of view.”*  *“And so I've seen new grad nurses doing someone's hair with like a steam iron that was diffusely psychotic and known to assault nurses. It was a dangerous situation.”*  *“I was aware of the fact that the nurse knows the patient, she dealt with him previously, he'd been there a while already. There was no early warning sign recognition, there was no - I just I found the nurse to be quite … didn't actually add anything to that situation. Finally, she pressed the alarm bell which was good that was presence of mind, but it was too little too late in my opinion, because by then me and one other house officer were actually involved in a physical struggle with the patient.”*  *“We were meant to meet the police at the clinic for the assessment and instead a decision was made that we wouldn't wait for the police because they were taking too long. So we went and we assessed him, just me and the mental health nurse without the police.”*  *“I worked in a community mental health team where all the offices had these alarms on cords. And I worked there for four months before I had a good look at the alarm. And when I kind of tugged on it, I found out that it wasn't attached. It was just, it was just like a cord with a button on the end and it wasn’t attached to anything!”*  *“In the inpatient unit where I am at the moment, there is actually… you can actually go to the desk and get a safety alarm. Yeah, that's what it is. And you can wear it around. Nobody tells you really or they might tell you once when you show up but when you actually go to try and get one they often don’t have one.”*  *“Working in outpatient clinics without robust security systems around or there's no security person or security alarm system is not functioning very well and stuff like that. It all makes us more vulnerable to assault and violence.”*  *“…sometimes I find your threshold and the nurses threshold can be a bit different.”*  *“There was a nurse on at night, but it was too busy for her to be in there with me.”*  *“And I wonder if – I don't know how the staffing kind of – everywhere is so understaffed you know… it's such an understaffed area that I think if we did have enough staffing to have more, you know, always have two person assessments, that would be a much better way to do things.”* |
| **The on-call environment is inherently fraught with danger** | *“Unfortunately the home visits were often kind of dangerous like that.”*  *“I guess kind of what I talked about - not having that support staff overnight, or on site.”*  *“And there's no one around in ED.”*  *“And I think, we need to somehow be standardising this acute call, the environments that we do those reviews in as well. I think it's silly to be going out to people's homes. I think it's ridiculous.”*  *“Usually we work in unsafe environments, especially in the community environment. Maybe inpatient is more structured and more kind of… but outpatient community settings are very unsafe, especially home visits.”*  *“I think out of hours, night-time, seeing people in their homes, in environments you can't control, you're not familiar with, is a real problem.”*  *“Because there was all this talk about they’re going to get a nurse to support you overnight, but who knows how long that will take?”*  *“I got to the car and turned around and saw that the ED charge nurse was walking away and walking back inside. So I was truly alone.”*  *“…with a lot of substance use…”*  *“Yeah so there was one main incident that happened when I was at work, I was doing a night shift. You’re by yourself, there’s no nurse on with you.”*  *“It was a night where we didn't have a backup register, so I was the only one on.”*  *“I think not being left by yourself to be in situations which does happen quite frequently. And I don't think high workload is an excuse because say if there is an incident, that's going to throw out the whole service anyway. So I think it's false economy to just try and spread people thin.”*  *“And I'd asked, I was happy to see her and I said to the nurse in ED that I wanted us to go together and to see her in an area that was you know, just allowed for a bit of room and safety and things like that for her and us. Particularly if she is now very psychotic and she's coming down from meth. And she wouldn't come and she went off, I think she went to dinner or something.”*  *“There’s been lots of times when someone has escalated while in the ED and security haven't been available. And that's been a problem.”*  *“Especially, I mean, if you think about the weighting of staffing, so 99% of staff work during the day, and then you just have sometimes just a reg, or a reg and a nurse overnight. And when do mental health presentations happen? They happen at night-time - because that's when people get sad and that's when people can't sleep. So that’s just kind of the weighting of the resources that we do have are a little bit illogical in my opinion. So I think that's a big factor as well.”*  *“But not having a clinician on site overnight – not cool. I don't feel safe with that. Particularly when we're on during the day, it's this big thing - you must have another person with you. And then suddenly, it turns 10 o'clock at night. It's like, oh, you're not going to have someone for 10 hours. I mean you're in ED, but that doesn't mean anything and you can’t always get the support that you want.”*  *“Whereas when I'm on call and I don't know them and I see some big dude - that's when I worry about my physical safety, because I don’t know them. I don't have a relationship with them. They don't know me.”*  *“I couldn’t get rapport with him.”*  *“I think psychiatric patients are inherently unpredictable, emotionally unstable and dysregulated. Quite frequently they lose contact with reality and they just lose impulse control.”*  *“You know, she was very forensicy, you know, a very kind of dysregulated woman.”*  *“Yeah. I think with other sort of comorbid things we see like substance use, people with forensic histories, we’re just more prone to seeing people who are maybe more violent.”*  *“And then it seems almost everyone has taken meth or polysubstance use and so I think those things make psychiatry dangerous.”*  *“But he got out of the car and just came straight over and gave me a good punch in the gut and walked off.”*  *“And then he said, ‘you better fucking leave or I'm going to kill you.’”*  *“I was worried that he was going to figure out where I worked and that he was going to show up and that he was going to show up when I arrived or left work and when there weren't many people around.”*  *“When actually a lot of the time I think there's honestly a really fine line where a home assessment is appropriate. And I think the second you think someone needs an admission and they're carrying risk then actually, we should probably be doing that in an ED because if nothing else, that means that we've got grounds to… we've got the ability to detain them and hold them. But also if the risk is at that level, then I do not necessarily want to be on ground that they know that I don't.”*  *“But I do think that when you are in the community late at night, often alone or the other problem is, mental health nursing is dominated by female staff so you end up with two females as a female trainee.”*  *“I've been in obviously more risky situations that I wouldn't have had prior to being a registrar such as home visits, after daylight hours and with people who are at a lot higher risk than I've dealt with previously.”*  *“I think on call and after hours work, I think a lot of it is difficult because of the system constraints and workload where like I've talked about before, we're needing to see people as soon as possible.”*  *“And then more on call, I think there's a variety in different areas of work about home visit policies and things but I think there's been some situations where I felt that there's been potentially dangerous situations at night, at people's homes, which were probably not very urgent and could have been left to later and things that could be avoided, when thinking about some of the triage, the quality of the triaging and things.”*  *“Our on call shifts are going into people's houses where they're… it's their environment and they’re unwell and we don't know what the environment is like.”*  *“Then, as I said, home visits I find I’ve been on a fair few that have just not sat right as you get there. And then afterwards you think actually that was really not good. I don't think I should have gone into that house but I was there and I was with the nurse and when we went in.”*  *“And he was also sort of acutely intoxicated with methamphetamine which obviously contributed to his agitation.”*  *“But I guess, the reality is that a lot of the clientele we do see on call and in the inpatient unit, have got acute psychosis and are abusing substances.”*  *“There was a narrow corridor leading out to the car. It wasn't the best decision for a place to do an assessment. The saving grace was that we were on the side of the kitchen table that was by the kitchen door.”*  *“The most danger I've encountered has been on call in home visits… If I was to go through the dangerous experiences, they've all been home visits, the most threatening ones to me.”*  *“I was seeing the person alone and this is in an emergency department where there is only the registrar, there is no other awake mental health staff members.”*  *“So actual on-site support when you're seeing someone on call. I mean, I almost always have someone with me, another clinician, but at night-time, I don't. And I find that's when I feel probably the most unsafe.”*  *“And so this often happens in crisis context where I might be asked to go see someone on my own…”*  *“And I think seeing people, crisis assessments on your own is quite risky.”*  *“I think drug use probably often contributes”*  *“And then I went to see them in the community on my own, like with a nurse, no police, no nothing. And as I'm knocking on the front door, I'm thinking is this proceeding as if this person has to firearms?”*  *“One particular instance when I went out to a guy with bipolar disorder and psychosis with a forensic history without police protection, who is known to be a kickboxer, known to have weapons and has, and I sat in his living room as he was floridly unwell and very agitated, with no protection at all whatsoever other than the small elderly female beside me. And that was a pretty threatening situation that I had to navigate carefully.”*  *“The room itself was tricky to navigate out of. I was in a couch as best I could, close to a door, but the way the house was designed, it would be very difficult to get out if he pulled a knife or an axe, which he had said he had in the house at some point. So it was very much… it was a threatening environment.”*  *“But he was a guy that was non-compliant with meds, forensic history, big, agitated, in his own home, which we didn't know. Dangerous.”*  *“So yeah, I would say not seeing people at home. And if you have to it's only like you must. And actually, I would say without a police escort you shouldn’t see anyone at home.”*  *“If you're sitting in someone's living room and it's two o'clock in the morning and they decide they want to assault you, you tell me what is possibly going to save you at that moment?”* |

**Theme two: Empowering trainees to address a sense of learned helplessness**

| **Learned helplessness and blame within a medicolegal context** | *"So you're actually put into situations as a junior doctor that you don't feel like you can push back on or control. So your safety is out of your own hands to some degree, because somebody else has done all that for you. But then if something goes wrong, that's suddenly your responsibility, it's your medico-legal responsibility."*  *“So as a doctor, junior or otherwise you have very, very little control over who you see, where you see them, what you see and any concerns you might have about your personal safety, are reasonably well fobbed off by these old experienced nursing staff.”*  *“And if we hear that we haven't been supported if we've been assaulted, then I think it makes people… it changes the morale and it changes how willing we are to raise something when it's a concern. So I can think of an example of a colleague being assaulted and then sort of not being supported when they had recommendations, like medical recommendations as a result of that assault. It just made the whole group of us feel like they don't like…like some kind of people in higher up management positions just don't care.”*  *“I guess sometimes I feel like if I bring up a safety concern, or if I say I don't really feel comfortable with this, it's sometimes brushed under the rug a little bit.”*  *“I think sometimes especially being more junior, I think when you're put in situations when you're interacting with people who are more challenging in terms of risk and feeling like often you're carrying that responsibility people are more, other clinicians and things are risk averse, trying to put that stamp, your doctor label on that.”*  *“So, yeah, I feel like if our kind of opinion on that was taken a little bit, a little bit more seriously, that would be good.”*  *“I feel like you have to go along with it. So I feel like if our opinion in the triage process was taken a little bit more seriously, I feel like that would…I don't know if it would actually improve safety or just help me feel safer.”*  *“I don't know whether part of that is the fact that I’m a reg and don't feel I have the authority to kind of bring it up further.”*  *“I feel like if I did absolutely say no and refuse and not go, that is an option, but you also don't want to be labelled as difficult.”*  *“And I think that we often end up with a culture that means that people feel pressured to put themselves in situations where it's against their better judgment.”*  *“Yeah, I think it was very disappointing when there was a group of registrars who we all raised our concerns about home visits at night time. And when it felt sort of that concern felt like it wasn't heard and something… that just needed to be… it felt like it just got pushed under the carpet and we were pressured to do it. And then we ended up… this was some years ago, getting the union involved and escalating it that way. But it was just disappointing that certain management people were sort of okay with us being at that level of risk when we weren't okay with it.”*  *“And yeah, sometimes it's just once the ball starts rolling, they've decided they're going to do a home visit and so they've contacted you and they're on the way out the door and you kind of have to be too.”*  *“And then the demand on your time, oftentimes you'll feel pressure to see someone without a nurse present which is dangerous for a bunch of reasons.”*  *“And I guess there is still a little bit of resistance, say on things like home visits if we do want to advocate for things like police presence and things if we do think there's elevated risk.”*  *“I've had several home visits where I was a much less experienced registrar and I questioned how necessary they were at the time. We kind of got browbeaten into doing them anyway…”*  *“There’s sometimes pressure to see someone quite quickly and things when maybe some time to settle and a good sleep and medications and an assessment following that will really make a big difference. But I think we're sort of pressured to try to intervene and just make decisions a bit earlier than maybe is appropriate and in that case, I think the risk definitely would of been mitigated if they were left for a little bit to settle rather than seeing them almost as soon as they got there due to the pressure of the bed situation.”*  *“… you're sort of more pressured from other clinicians or the system or the service to sort of discharge them which I find quite difficult and sometimes I’ve had to take home with me people that I feel quite uncomfortable about their risk. You get a feeling about them, but you know, you're having to make a decision because of the resources”.*  *“But also then the bar for safety has been set. These people have learned the bar for safety, particularly in crisis teams, it’s set quite low. So for new regs, I would imagine you'd feel, it would be tough to request more than is offered and so you're going out to these situations that are dodgy.”* |
| --- | --- |
| **Self-awareness in culturally safe practice** | *“The thing which is probably kept me safe is the fact that actually, I seek to level power in every interaction. And it means that someone is relating to me as a human, rather than a doctor that's going to put them under the Act. And I think it's a much more pleasant way to practice but I think also people respond to it and I've consistently found the people that you'd normally expect you'd have difficulty with, if you do that they're not the ones you have difficulty with. So I think that surprisingly actually, culturally humble practice and basically not being afraid to go ‘yeah, I don't know I'm not the expert. This is what I do know’ and kind of staying in your lane. I think actually keeps you really safe.”*  *“I think having cultural support more readily available and it should be a much more important aspect of care on call.”*  *“I think having real… I've yet to… I have to be honest I have yet to attend a cultural learning day where they give really practical moment to moment of stepwise advice on how to engage these patients and manage safety.”*  *“So if we can do as much as possible to have as much cultural liaison input so that we can work as collaboratively with the clients rather than it being felt as us and them.”*  *“I'd like to have more cultural knowledge from other cultures.”*  *“I’m more thinking about the safety for the other person, which I guess by extension is thinking about my own safety because if that person is settled and is feeling heard and validated, and it's within what they expect within their cultural context, then they are less likely to get agitated and have a go at me.”*  *“And so, yeah just even thinking about the historical traumas that have happened to Māori and when they're in the throes of a psychosis and put in the establishment that kind of may evoke a lot of that intergenerational trauma or distrust and mistreatment by the establishment institution and it can be understandable in some of those frameworks how these kinds of behaviours gets opposition and may be heightened in those contexts.”*  *“They're probably in an unfamiliar environment and so anything we can do to provide that familiarity, whether it be bringing things in from home that make them feel comfortable, photos, any kind of like… just objects that they hold dear, that can just help them feel grounded and things. Obviously the family input is a really important aspect of that too.”*  *“Which is with patients what I try to make clear is that they're an expert in their own lives. I have some experience around what goes wrong for people and what that looks like. So to understand what's going on for them and to make a plan we need their expertise. We need my expertise, and we'll make a plan together. And it's really important to my practice to take as much of that power imbalance out as I can, which basically means taking steps to minimise my own power as the health care provider and taking steps to maximise theirs. And I think that this is true of anyone. I don't think it's limited to Māori and Pacifica. And I think you can do things which basically, whenever I've taken that approach, it's gone well, and it means that I kind of go ‘look if there's anything I can do to make you more comfortable’, which is whether that's we do a karakia, we do a waiata, we do whatever. Or you know, if you're Indian Sikh Muslim, we pray or whatever. Then let me know, and we'll do it. And I think it takes out the assumptions around all Māori want a waiata, all Māori want a karakia.”*  *“But I think the key thing is that I’m not Māori, I'm never gonna be Māori. Māori are experts in Māori. And what I can do is I can sit back and go look, I'm not an expert, but I'm happy to help.”*  *“And on top of that, I guess, extending beyond just cultural sort of competence is cultural safety and thinking about the various unconscious biases and stigma that we may hold and just, I guess, cultural supervision or something”*  *“I think that actually, the most useful thing is probably the broader strokes of the cultural training, the Māori and Pacifica stuff and particularly around this idea of cultural humility, and recognition of power imbalance.”*  *“The presence of that in the inpatient unit and forensic unit was very limited and absent.”*  “*So I've had a lot of times where it's been a Māori whanau and there was no Māori cultural support available.”*  *“I'd like to have more cultural knowledge from other cultures.”*  *“It's not very available to be honest.”*  *“I think there's no doubt, there is no doubt that if you can have whanau present like I said earlier, I think if you can… I think the really important thing with Māori is, is to even the playing field. There's a sense that they feel disempowered and when they're in an appointment with a doctor or some white highly trained professional, I think the playing cards are stacked against them.”*  *“So that's definitely in mind and also the fact that a lot of people in different cultures we work with, they don't align with the medical model, which is what is more our focus as psychiatry registrars and training psychiatrists. So I think that’s in mind when people are not collaborating with our plans and what decisions on disposition and things, that’s definitely in mind.”*  *“That sort of support is unfortunately, an area that needs improvement now where I am, it is lacking which is unfortunate.”*  *“Have I used it? When possible, but it's often not available. Again, a handful of times where it’s not been available, but when possible, I'd kind of utilise any cultural support. Or if I’m seeing a Māori patient, bring a nurse, when possible, but it seems a rare occurrence that it’s on hand when you need it.”* |
| **Forewarned is forearmed; Managing workplace violence as a core component of**  **training** | *"So for trainees particularly I think that we do a really big disservice in onboarding people. I think that a lot of the new registrar's end up basically doing shifts on their own. And I don't think that they have that benefit of being shown the ropes by people. It's a bit of a baptism of fire.”*  *"I guess to support the, the kind of the clear training about what we can do and what to do when we're in that situation. I mean sometimes it may be obvious, but I think just to have it formalised on a bit of paper or in a training module, or teaching. I think it will give people more confidence to assert that."*  *“Like I didn’t need to probe him that much for information when he was in the state he was. So I guess my lack of education, lack of support.”*  *“It could also maybe even be part of an EPA or something like that. I'm not sure - just to make something a little bit more formalised rather than just learning on the go often from doing things that you probably shouldn't be doing.”*  *“I had them back to back so I didn't have any break.”*  *“I didn't think the SPEC training that I did was particularly helpful.”*  *“What it did do though, was give me cause for thought about the next time and I thought to myself, gee, this is a terrible way to learn. It's a terrible way to learn smarts around workplace safety - to actually have to experience it rather than.. I mean it’s just terrible.”*  *“The fact that we do bend rules often and then providing training for the fact that we bend rules, would be much better than pretending we don't bend rules and giving us kind of…poor overall safety training.”*  *“I think also just how new and naïve I was, that I could handle that on my own I guess. I want to say I hadn’t learned the lesson yet, but I shouldn’t have learnt it this way. I just wasn’t properly aware of how much information I needed to take on to the next step and get myself to a safe place. Like I didn’t need to probe him that much for information when he was in the state he was. So I guess my lack of education, lack of support.”*  *“I think, you know, some of the policy stuff that we've been doing, I think should hopefully make a difference in terms of kind of just actually making sure that we've got procedures for escalation, but all of its around, I guess first is around the education side of things and ensuring that people actually have adequate support to learn.”*  “*I guess maybe more teaching on how to approach tough situations. I’m not sure if we really ever did get teaching on that. Especially as first years. Cause in the course I did, the whole first day was challenging incidents.”*  *“But it was a bloody battle to get any kind of training. It took six months to get in. So I think more easy accessibility to these courses.”*  *“I think it takes like such a lot of mental energy to do a really good assessment with really good rapport, really picking up on all those micro expressions and like, you know, it's just very mentally, a very intellectually demanding task. And so if the registrar's are saying, you know, this isn't working in regards to rostering and things. I think that that's also important because I think when people are irritable, when people are tired, when people are burnt out, the patients, some patients are extremely sensitive and they can pick up on that, they can be offended. Things could be said that could offend them or make them feel like they're not important enough or and then, like if the rapport breaks down, that's when things are misinterpreted and that's when things can go wrong. And so I think that we really need to be supported to be at the top of our game as much as possible like there are factors that are not allowing us to perform because like it's a really high performance role that we're in.”*  *“We don't have a lot of robust frameworks in psychiatry.”*  *“… psychiatry is a funny specialty because there's only a few specialties in all of medicine and surgery that you actually don't have any true exposure to as a house officer. Psychiatry is one of them. And there might be a couple of others maybe, quite subspecialised areas that you could honestly say, as a house officer, you don't get any practice.”*  *“And so to me, psychiatry, the college, the local training schemes, they should be aware of the fact that they're unique in that everybody here on day one of their registrar job has never conducted a psychiatric interview before. Usually, that's usually the truth. And so they've never conducted an interview and they've got no clue about how to conduct themselves. They've got no clue about safety. They've got no clue about any of these things. They've had almost no exposure to psychiatry. And I think the college therefore should have some sort of mandate around what happens to new trainees because I think it's quite unfair. I think it's quite unfair to be expected to formulate a patient on day one of your registrar training when you've never done it before. And safety's the same. You've got no idea what to ask or not ask a psychotic patient. You've got no idea what early warning signs might look like. You've got no idea how to set yourself up in the room. You've got no idea whether to trust the nursing staff or not when they tell you to go and see somebody. You don't know. You’ve never been there before. And there's no, there's no lead in. There's no lead in. Zero lead in.”*  *“No adequate training in safety measures in place for clinicians and trainees.”*  *“… because I feel like we're often just like flying by the seat of our pants and using our own judgement to figure out how much kind of, of that behaviour we do and don't take. Maybe more protocol around it, about what we are expected to do.”*  *“As I’ve said, it’s all been very informal learning on the job. What I am and aren't comfortable with and learning from the mistakes almost more usually than what's gone right. Yeah. So I wouldn't say I've had any formalised training or resources and I've never really looked at it. I would have liked some I think.”*  *“But how this often transpires is ineffective safety training that feels more like a tick box than actual care for reg safety.”*  *“The training we are provided with it's not enough at all. It’s almost no training. We receive a lot of training about risk assessment, risk management usually related to self-harm, but we don't receive training for violence as much as self-harm and suicide. Because all the focus is on suicide and self-harm, but what about our safety? The curriculum needs to reflect the reality that violence is very common.”*  *“A framework of some sort that says, this is how we're working through it. We're prioritising staff safety, and this is what it looks like. And we have to balance that against service delivery.”*  *“I think consensus around precautions that could be taken and all regs being versed in it. And it just being kind of like a general consensus. Just like we know the medication routines. We know the interview routines. Having a safety routine that's followed each time and installed within us, would be really helpful from the first part of training and not this fluffy physical training we get - it's just absolute nonsense. It's just nonsense. It's useless. So I think more street savvy training both in physical engagement and also cultural engagement would help a lot.”*  *“We get training on a lot of things that's less useful. I think incorporating it even in the mandatory reg training like the teaching. To have a teaching session, for instance, on lived stories of when it's gone wrong and the lessons learned”* |

**Theme Three: Conflict embedded within the unique nature of psychiatry**

| **Resistance to a psychiatry shaped by stigma and historical abuse, evoking**  **violence.** | *“And the other side of that is the public perception, which is I think we've previously done some pretty shit things. And I think that the public have not yet forgiven mental health for this. And in some pockets we’re seen as people that … we’re seen as the enemy and people that take your take your rights away. I think it's a deserved reputation, but I think actually we need to take steps to apologise and rectify that. But other areas of medicine don't have that reputation. They're seen as people that sweep in and save your life.”*  *‘We’re the ones that I guess are labelling them as unwell and forcing medications on them. I guess compared to other specialties, people are the majority of the time grateful for the help they have received.”*  *“…it sort of seemed like they as a family had stigmatising attitudes towards mental illness…”*  *“You tell them that they’re unwell and they’re going to get pissed off.”*  *“I guess on my last set of nights, my last patient on my last set of nights was this Mongrel Mob gang member who was manic... he was pleasant until I started telling him that I am concerned he is unwell and might have to come to hospital.”*  *“… a lot of the trauma or experiences that people have had in healthcare or the mental health system…just exacerbates that mistrust.”*  *“And I think it means that we start on a back foot with the public that are already angry at us, already expect us to be shit and fail. And kind of want to, you know, abuse their rights and things like that, which I think it's a public image issue, but I think it contributes to violence.”*  *“And then I also think that psychiatry itself has this enormous sort of stigma attached to it. And I think that when you come along as a psychiatrist or a mental health doctor and you are essentially telling somebody you think that they are mentally unwell, there is a lot of natural resistance to that and I think it stirs up a lot of really intense feelings, not just in patients, also in the family members of patients and a wider circle.”*  *“And they're already wound up and they already don't want to talk to you and then they're in a position where you're forcing them to do something they don't want to do and quite naturally they feel aggrieved.”*  *“I know how difficult it is to be a client in the mental health system and the past traumas they must have had in the system.”* | |
| --- | --- | --- |
| **In the line of fire; Restrictive contexts invoking combative response** | *“So there’s sort of those patient factors, just the nature of the conditions and also the fact that you know, with most other specialties the patients are voluntary and actually want to see the doctor.”*  *“In theory, we call the police, we ask to see the police. There's no coordinated response between the police and us so we're stuck waiting for the police while risk is escalating in the community, and in the end, we put ourselves at risk to try and de-escalate community risk of having a psychotic person.”*  *“Defensive medicine means that we often end up putting people into an inpatient unit and restricting their rights who, ultimately it's not going to be for their good.”*  *“And so the only option really was to start t*he *Mental Health Act and detain him.”*  *“I think there have been one or two occasions in the emergency department, often this is where people have often come via police or forcibly, and they may not have had any treatment or any medications to help with sedation.”*  *“I mean, I guess some of the factors I've touched on before - the way the Mental Health Act is set up and the coercive nature and the way that inpatient units or mental health facilities run – I think it can speak to a lot of the trauma or experiences that people have had in the healthcare system or the mental health system and just exacerbate that mistrust and things.”*  *“I think friction with the police is another issue and police not being open to uplifting people when they probably could do.”*  *“I got called to assess someone at a house where the police had refused to bring him in because they didn't think they had grounds and on site the guy was brandishing a knife surrounded by four police officers in stab vests and the police tried to convince me that I needed to go into the middle and talk to him.”*  *“There's lots of times where they've, they claim it's voluntary, but you talk to the patient, they clearly didn't come willingly and it was 109. And I really worry about the legalities and the ethics, but the knock on effect is that riles people up. And they're already wound up and they already don't want to talk to you and then they're in a position where you're forcing them to do something they don't want to do and quite naturally they feel aggrieved.”*  *“I think that the police mental health front is a real problem. We need a coordinated - they trialled, they piloted a coordinated service - I don't know exactly how that went but it makes perfect sense to me that out of hours call needs to be better coordinated with either our own security service or police.”*  *“Yeah, so there was one client who was brought in by the police, he didn't want to be there.”*  *“The police had apparently section 109’d him, but when they arrived and they just left. Like there was no swap out with security. There was nothing really. They just left and kind of left him in the room.”*  *“Even the police officer told them, could you take him in your own car, we're short staffed and I just said no. I just told the police officers that's not acceptable and we're not filling in for your staff gaps, we have our own.”*  *“You know, every third word being ‘fuck’, accusing us of all sorts of things – keeping him against his will, which, you know, he was under the Mental Health Act.”* |  |
| **The emergency department is not fit for purpose** | *“And also that room, it’s the room where they put all the people that they are most concerned about risk of violence – and the door it locks. When you shut the door from inside the room, you have to swipe your swipe card to unlock it….The swipe access point was right by where the patient was sitting. So I would have had to either shut the door and have that delay if you want to get out, or you have to leave the door open. It’s a public ED corridor. So what I mean, it's off to the side but there is another patient just across the corridor.* “  *“Yeah the spaces are not often well designed for our purposes.”*  “*I think that even though it was close-ish to the nursing station, it was one of the single bedded rooms with the – I can’t recall but the door that sort of closes. I think it was during COVID where that room we had for psych wasn’t available because it was being used as a PPE room. But even so, that room would have been even more sort of away from staff.”*  *“There is a tendency of once they've been referred to psych that kind of protection part has gone, like they might have security there, but they’re just keeping less of an eye on things.”*  *“Or as I was saying in terms of staffing and spatial design, whether there's even better spaces in ED to be undergoing those assessments, but obviously in the inpatient unit too.”*  *“I think not necessarily having safe places to assess and ongoing friction with ED about bringing people to ED for an assessment.”*  *“So this is where often they're very acutely agitated and the environment is not conducive to… it’s very high stimulus. It's a very difficult place to be and so this is often where I find people are more agitated.”*  *“I think there was a trial during COVID when the magical money tree suddenly sprouted and we had money when we were able to see people in the community basis instead of diverting them to ED and instead of them having to wait in ED. And the patients thought it was great.”*  *“…in ED settings people keep verbally abusing, swearing and stuff.”*  *“There’s been lots of times when someone has escalated while in the ED and security haven't been available. And that's been a problem.”*  *“So ED, although they're pretty well set up because unfortunately they also get a lot of violence, not just from our psych clients. There is a tendency of once they've been referred to psych that kind of protection part has gone, like they might have security there, but they’re just keeping less of an eye on things.”*  *“But you know, it’s this room where there's not many windows and it's just the one door that was openable, one exit and then he just got really worked up within a few seconds.”*  *“We were isolated in the room, it was in ED. I doubt whether they would be able to attend quickly.”*  *“We're seeing a lot of people who are waiting in ED quite acutely unwell, with mental illness and the wait times are long and then if they are needing - the ED is just not a very conducive environment for that and so we try to see them as quickly as possible, but the ED is quite stimulating, noisy, or the lights and sounds and not a lot of privacy. So we try to, we want low stimulus, but it's just the opposite.”*  *“And then, because there's not enough beds on the inpatient units, often these people might have been waiting a few days and so that can really escalate ones risk. When and you know, they're obviously not getting the level of nursing input and psychiatric input they would be in a psych unit.”*  *“… you're much more likely to get into abusive interactions with other staff members because they're so frustrated at the volume of mental health problems coming through the hospital and their natural inclination is to take it out on you as the mental health physician.”* |  |
